# Trace Elements in Gluten-free Pastas and Flours from Markets Located in the Las Vegas, Nevada Area

**DOI:** 10.5539/jfr.v8n5p59

**Published:** 2019-08-14

**Authors:** Christopher J. Collumb, Adam A. Delelegn, Giavanna M. Fernandez, Amanda C. Hudson, Kendra W. Kimberley, Douglas B. Sims, Douglas J. Walton

**Affiliations:** 1 Department of Biological Sciences, College of Southern Nevada, North Las Vegas, NV 89030, USA; 2 Department of Physical Sciences, College of Southern Nevada, Las Vegas, NV 89146, USA

**Keywords:** gluten-free foods, nutrients, metals, celiac, pasta, flour, Las Vegas

## Abstract

The popularity of gluten-free foods has been increasing across the United States and abroad. A significant reason for this trend involves marketing efforts targeted towards individuals seeking to avoid the consequences of celiac disease or a perceived gluten intolerance. Many gluten-free food products originate in regions of the world where irrigation with metal-contaminated waters is common. Calcium, Fe, Mg, Ti and Zn were detected at various levels across all foods products. Cadmium was detected in 96.8% of U.S. and 54.5% of Asian gluten-free foods with gluten containing foods above reported averages (216 μg kg^−1^ Cd); as was Co (140μg kg^−1^) in 48.4 % of U.S., 72.7% of Asian gluten-free foods, and 40% of the gluten containing foods; Cr was in 54.8% of the U.S., 72.5% of Asian gluten-free foods, and 100% of gluten containing food products; while Ca, Fe, Mg, Ti and Zn were greater than 10,000 μg kg^−1^ with Ba, Cd, Co, Mo, and Ni above reported averages. Finally, trace metals were more commonly detected in the gluten containing foods overall. It was found that trace elements were more commonly found in the gluten containing products; however, none of the higher than expected levels pose a significant health risk to consumers.

## 1. Introduction

Gluten is a protein found in breads, pastas, and products of wheat, rye, barley, and triticale. According to literature, the proteins glutenin and gliadin are the main products of gluten with gliadin responsible for most of the negative health effects (Basha et al., 2014). Gluten helps food maintain its shape while also binding it together (Basha et al., 2014). For person with a gluten allergy, gluten is known to cause small intestine irritation, among other symptoms, in sufferers of Celiac disease, a genetic based immune disorder (Basha et al., 2014). According to other researchers, celiac disease is characterized by an immune response of the T-lymphocytes in the small intestine to gluten peptide bonds (i.e. Basha et al., 2014, Howdle, 2003, Miedico et al., 2017, Orecchio et al., 2014, Thompson, 1999). While medically debated, many people claim to be gluten sensitive and report allergic response to its protein.

Research suggests that celiac disease occurs in approximately 1% of the general population (Niewinski, 2008, Thompson, 1999, 2000, Thompson et al., 2005). In addition to sufferers of celiac disease, many people avoid gluten because of a wheat allergy or they believe they have a gluten intolerance, despite there being limited medical evidence that such a medical disorder exists (Elli et al., 2015, Catassi et al., 2013). Current studies indicate that celiac disease is increasing across all age groups, especially in the senior population (Curiel et al., 2014). The only currently available treatment to this disease is a strict gluten-free diet, which can lead to the restoration of the atrophied intestinal villi (Curiel et al., 2014, Niewski, 2008).

The consumption and/or baking with gluten-free products have become much more prevalent due to product availability (Orecchio et al., 2014). Gluten-free consumers can purchase premade gluten-free products such as breads, pastas, flour, and desserts. Today, many foods contain gluten-free starch and flour made of corn, potato, tapioca, or rice. Gluten-free baking mixes and flours are becoming accessible to consumers so that they can bake their own breads, pastas, cake, and other foods, which in turn alleviates the need for them to consume a diet consisting of mostly raw and green foods (Orecchio et al., 2014).

Trace metals have a vital role in biology and are essential micro-nutrients that play an active role in biochemical functions of living organisms (Miedico et al., 2017, Orecchio et al., 2014). While some metals like Al, Cr, and Fe are essential to biological functions at low levels, they can be toxic at higher levels. Other metals (i.e. Cd, Pb, Se, V) do not occur naturally in the body, and therefore, their presence is usually the result of ingestation from foods. Exposure to such metals can be harmful to human health, especially in children and the elderly who tend to be more sensitive to these toxins (Miedico et al., 2017).

Gluten-free food products may contain higher and lower levels of trace metals depending on the product or where they are grown in the world (Orecchio et al., 2014, Thompson et al., 2005). Studies have identified that Cd-contaminated wastewater from industrial sites have been used in Asia’s agricultural fields (Takagi et al., 2004, Vahter et al., 2007, Yamagami et al., 2006). Consuming foods containing Cd over a lifetime can result in a mild to severe mitochondrial dysfunction called Itai–itai disease (Vahter et al., 2007, Yamagami et al., 2006, Inaba et al., 2006). Extended intake of certain pollutants (i.e. I, Pb, As, Cd, Hg) can lead to other disorders in the gastrointestinal tract. The World Health Organization has determined acceptable levels of trace metals in food in cooperation with other international authorities for the protection of human and environmental health (Yamagami et al., 2006, Inaba eta al., 2006).

There are several gluten free cereal products that are primary ingredients for these foods such as rice (Oryza sativa), maize (Zea mays), sorghum (Sorghum bicolour), and buckwheat (Fagopyrum esculentum Moench) according to Orecchio et al. (2014). While there is nutritional value in these foods, they are only part of an overall balanced diet. Buckwheat, for instance, is known as a source of protein, amino acid, vitamins, dietary fiber, minerals, and essential trace elements (Orecchio et al., 2014, Howdle, 2003). Buckwheat; however, has lower concentrations of essential metals (Zn, Fe) when compared with wheat (Orecchio et al., 2014, Vahter et al., 2007). Moreover, the grinding processes to turn such products into flour can also alter the nutrient composition of the food product (Thompson, 1999).

Trace metal concentration in mainstream food products has been well studied however, little information exists for gluten-free foods (e.g. Aziz et al., 2015, Orecchio et al., 2014, Ertl and Coessler, 2018, Parra-Orobio et al., 2017). This research measured trace metals, both toxic and non-toxic, in gluten-free foods (n =42), and the daily intake concentrations. A limited number (n=5) of gluten containing foods were analyzed for comparison. The overall study objectives were to evaluate the levels of trace metals (Al, Ba, Ca, Cd, Co, Cr, Cu, Fe, Mg, Mn, Mo, Ni, Ti, Zn) found in selected gluten-free food products (i.e. pastas, flours) purchased in the Las Vegas, Nevada (U.S.) market. This study will: 1) identify crustal (Al, Ca, Fe, Mg, Zn) and toxic trace metals (Ba, Cd, Co, Cr, Cu, Mn, Mo, Ni, Ti) in gluten-free food products; 2) determine the levels of trace metals in specific gluten-free product types; and 3) if possible, evaluate the level of exposure (i.e. chronic exposure) to consumers of these foods in the literature and discuss possible effects due to low-level chronic-long term exposure.

### 1.1 Metals in the Food Chain

It is known that toxic elements in foods can be from both natural and anthropogenic source (Orecchio et al., 2014, Baker et al., 2018). For instance, Orecchi et al. (2014) determined that metal contamination of food products is related to the soils it grows in and rarely the manufacturing (milling) process. Other researchers have examined the uptake of metals (e.g. Cu, Ni, Fe, Co, Zn, U, Pb) in vegetation, animal tissues, and the transfer of said metals via the food chain (Miedico et al., 2017, Kipp et al., 2009, Clemente et al., 2005). High levels of Pb tend to be present in broad leaf plants (Elmleaf, Blackberry and *Rubus ulmifolius*) grown in contaminated soils as documented by Clemente et al. (2005). The bioaccumulation of As, and resulting phytotoxicity, was observed in areas that had several metal-tolerant plant species (e.g., *Angophora floribunda, Cassinia laevis*, and *Chrysocephalum apiculatum*) that colonized the periphery of contaminated sites or were irrigated with contaminated waters (Clemente et al., 2005, Han et al., 2006).

It is known that high levels of metals are possible in grasses, melons, coconuts, fruits, and even in rice, corn, and wheat where effluent from factories or mining is utilized for irrigation (Nicholson et al., 2003, Vazquez et al., 2008, Wang et al., 2007). Crops grown in the mining region of the Sierra Madrona Mountains of southern Spain showed higher concentrations of trace metals (Pb, Zn, Cd, Cu, As and Se) (Orecchio et al., 2014). It has been reported that higher concentrations of trace metals in vegetation were more prevalent in crops like rice and wheat where irrigation originated from polluted waters (Reglero et al., 2008).

It is known that contaminated crops affect the food chain and ultimately human health (Inaba et al., 2006, Yamagami et al., 2006). Cadmium contaminated wastewater from industrial sites was used for growing rice in Japan and ultimately impacted local health (Takagi et al., 2004, Vahter et al., 2007). Studies found that residents in the area who consumed 3.1 g to 3.8 g of Cd in rice over a lifetime developed mild to severe mitochondrial dysfunction called Itai–itai disease (Inaba et al., 2006). The source of this disease was found to be the direct result of Cd-enriched wastewater discharged to local rivers, and subsequently used in the rice fields. The bioaccumulation of Cd in the tissue of local residents affected their tubular epithelial cells after 80-weeks of exposure by ingestion of the contaminated rice (Nation Academy of Science [NAS], 1973).

## 2. Materials and Methods

Forty-two (42) samples of gluten-free foods (pastas, flours) and five (5) gluten containing foods (pastas) were purchased in Las Vegas markets in June 2018. Products (“samples”) were representative of what is available in the Las Vegas Valley with 31 samples manufactured in the U.S. and eleven (11) imported from Asia. The five (5) gluten containing samples were manufactured in the United States (U.S.). While manufacturing location was known, the agricultural source of the plant material was not. Each sample was homogenized with an acid washed mortar and pestle, immediately sub-sampled and digested with hot aqua-regia acid to reduce degradation. All gluten-free samples were prepared in triplicate, digested, and analyzed per USEPA methods as described below. Data was evaluated for standard deviation (σ), mean ($\bar{x}$), minimum (min), and maximum (max) values seeking patterns within each group of products (i.e. gluten free vs. gluten containing) and across groups.

### 2.1 Sample Preparation and Analysis

Forty-two gluten-free and 5 gluten containing samples (n=47) were purchased from local markets and analyzed for trace metals (Al, Ba, Ca, Cd, Co, Cr, Cu, Fe, Mg, Mn, Mo, Ni, Ti, Zn). Approximately 1.0g (±0.001g) of homogenized food material (gluten-free [e.g. rice, corn] and gluten containing [i.e. wheat]) was processed according to USEPA Solid Waste 846 (SW-846) protocols, including quality control (USEPA, 1997). Fourteen trace elements (Al, Ba, Ca, Cd, Co, Cr, Cu, Fe, Mg, Mn, Mo, Ni, Ti, Zn) were extracted using USEPA method 3050B (hot aqua-regia acid digestion) followed by USEPA method 6010B for inductively coupled plasma-optical emission spectrometer (USEPA, 1997). Instrument calibration consisted of 6 points across a concentration range (including a blank) and fitted by linear regression with >0.995 R^2^ (USEPA, 1997). Instrument integrity was verified with a certified reference sample purchased from a USEPA certified supplier with the sample falling within certified windows in order to meet requirements. Samples were analyzed in triplicate with a required relative standard deviation (RSD) of less than 20% to qualify as acceptable (USEPA, 1997). Method detection limits (MDL) are defined as 3 time the standard deviation (σ), where the level of the analyte approaches zero (0). Limit of quantitation (LOQ) is defined as 10 times σ with an uncertainty of ~ 30% at the 95% confidence level.

## 3. Results and Discussion

Trace metal concentrations (Al, Ba, Ca, Cd, Co, Cr, Cu, Fe, Mg, Mn, Mo, Ni, Ti, Zn) for 42 gluten-free and 5 gluten containing food samples (n=47) are presented in Table 1 and reported in μg kg^−1^, dry weight. Table 2 shows statistics for all of the data in three separate formats; overall data, U.S. gluten free, Asian gluten free, and gluten containing foods of pasta and flour. Samples were processed in triplicate utilizing USEPA quality control measures for accuracy and averaged for reporting purposes. Aluminum was detected at 400 to 6750 μg kg^−1^ (mean ($\bar{x}$) of 1754) across all products with a U.S. market gluten-free foods $\bar{x}$ of 1708 μg kg^−1^. Asian gluten-free foods were 550 to 3000 μg kg^−1^ ($\bar{x}$ =1736 μg kg^−1^) while gluten containing foods ranged between 1450 and 3350 μg kg^−1^ ($\bar{x}$ = 2080 μg kg^−1^). U.S. market foods were highest in samples 7 (6750 μg kg^−1^ [pasta]) and 23 (5900 μg kg^−1^ [flour]). Asian foods were highest in samples 45 and 47 measuring 2150 and 3350 μg kg^−1^ Al, respectively, with 850 to 3.8 × 10^4^ μg kg^−1^ reported by other authors (Loska and Wiechuya, 2003, Yokel et al., 2005).

With Al at a level of 6750 μg kg^−^1, it is important to understand that other studies have established that oral exposure to Al is not considered harmful as it is poorly absorbed and is excreted through feces and urine (Finberg et al., 1986). It has been reported; however, that ingestion of high amounts of Al can lead to nausea, mouth and skin ulcers, skin rashes, vomiting, diarrhea, and arthritic pain (Jaishankar et al., 2014). These symptoms are generally mild and short lived although people with renal disease, especially children, are at risk of developing Al toxicity (Finberg et al., 1986, Jaishankar et al., 2014). Finally, the Food and Drug Administration (FDA) has determined that aluminum used as food additives, are generally safe; however, they have set an exposure limit for Al at 0.2 mg L^−1^ in bottle water according to ATSDR (2008).

Barium was detected at 350 to 1.2 ×10^4^ μg kg^−1^ across all samples ($\bar{x}$ =2606 μg kg^−1^). The U.S. gluten-free samples ranged between 500 and 1.2 × 10^4^ μg kg^−1^ ($\bar{x}$ =2494 μg kg^−1^) while the Asian samples ranged from 350 to 4050 μg kg^−1^ ($\bar{x}$ =1477 μg kg^−1^), and gluten containing foods from 3700 to 9700 μg kg^−1^ ($\bar{x}$ = 5790 μg kg^−^1). Literature has shown cereal products (both gluten and gluten-free) tend to encompass higher Ba levels such as bran flakes at 3900 μg kg^−1^ (Mertz, 1986). Current Ba data falls within stated ranges although 11.9% of samples exceeded Mertz (1986) average in samples 14 (7100 μg kg^−1^), 19 (6800 μg kg^−1^), 23 (12100 μg kg^−1^), 28 (9000 μg kg^−1^), and 37 (4050 μg kg^−1^). Data also falls within stated ranges reported by Millour et al. (2012).

Although our findings indicate that the highest level of Ba was detected at 1.2 ×10^4^ μg kg^−1^, according to the ATSDR (2008), Ba toxicity depends on its solubility in water and bodily fluids. Insoluble barium salts are nontoxic, whereas the barium ion (Ba^2+^) and compounds that can dissolve in water or can be diluted in hydrochloric acid, such as chloride, nitrate, carbonate, and hydroxide, are toxic. The predominant effect of barium toxicity is hypokalemia that can result in ventricular tachycardia, hypertension and/or hypotension, muscle weakness, and paralysis (ATSDR, 2008).

Calcium ranged from 1.4 × 10^4^ to 2.09 × 10^6^ μg kg^−1^ with an overall $\bar{x}$ of 3.55 × 10^5^ μg kg^−1^. Gluten-free products from the U.S. markets ranged between 1.4×10^4^ and 2.09 × 10^6^ μg kg^−1^ ($\bar{x}$ = 4.24 × 10^5^ μg kg^−1^) while the Asian samples were between 6.5 × 10^4^ and 3.36 × 10^5^ μg kg^−1^ ($\bar{x}$ =1.4 × 10^5^ μg kg^−1^), and gluten containing samples ranged between 2.07 × 10^5^ and 6.5 × 10^5^ μg kg^−1^ Ba ($\bar{x}$ = 3.9 × 10^5^ μg kg^−1^). It has been noted that Ca in gluten-free foods ranged between 5000 and 1.68 × 10^6^ μg kg^−1^ and was higher for gluten containing foods (Orecchio et al., 2015). Prolonged exposure to Ca is a concern as calcification in the kidneys (nephrocalcinosis) can lead to the formation of nephrolithiasis; i.e. kidney stones (Patel and Goldfarb, 2010, Ross et al., 2011). The current study falls above Orecchio et al. (2015) established ranges for the U.S. market gluten-free foods and therefore, may pose an issue with chronic exposure.

Calcium is vital for normal body functions and studies have established that it is difficult for a normal, healthy adult to consume excess Ca by diet alone (Patel and Goldfarb, 2010, Ross et al., 2011). If Ca toxicity (e.g. hypercalcemia) is an issue however, signs may include anorexia, weight loss, polyuria, heart arrhythmias, fatigue, and soft tissue calcification (Patel and Goldfarb, 2010). Other authors have found that tissues most at risk of calcification include vascular tissues, lungs, and kidneys. Additionally, calcification of the kidneys (nephrocalcinosis) may lead to the formation of nephrolithiasis (i.e. kidney stones) according to Patel and Goldfarb (2010) and Ross et al. (2011). Studies have established that children under the age of 4 have an Upper Intake Level (UIL) of Ca that various by life stages. For instance, infants 0–6 months in age have an UIL of 1000mg/day while the UIL in adults >51 years is 2000mg/day (Patel and Goldfarb, 2010, Ross et al., 2011). This study shows that the gluten free and gluten containing have levels far below the mg/day intake that would produce damage.

Cadmium ranged from 150 to 600 μg kg^−1^ with a $\bar{x}$ of 278 μg kg^−1^ across all products. The U.S. gluten free foods ranged between 150 and 600 μg kg^−1^ ($\bar{x}$ = 270 μg kg^−1^), Asian products between 250 to 500 μg kg^−1^ ($\bar{x}$ = 333 μg kg^−1^), and gluten containing products between 200 and 350 μg kg^−1^ ($\bar{x}$ =260 μg kg^−1^). It is worth noting that 45% of the products from the Asian markets contained no detectable Cd (samples 33, 34, 36, 39, 40). Other studies have established a range for Cd in plant materials including those used for food stocks to be 2 to 216 μg kg^−1^ (EC, 2006, Skrbic et al., 2013). Many of the samples in this study were above this range. With almost 55% of U.S. market foods, 36% of Asian, and 80% for gluten containing foods above the established range. Moreover, six samples (samples 5, 21, 26, 27, 32, 41) were 1σ above the $\bar{x}$ (379 μg kg^−1^) with gluten containing foods falling below.

Data show that gluten free and gluten containing foods have levels far below the mg/day for Cd intake that would produce harm. However, it is important to state that other studies indicate that soluble Cd compounds of SO_4_^2−^ and Cl^−^ salt forms have been shown to cause both acute and chronic intoxication (Jaishankar et al., 2014, Tchounwou et al., 2012). Moreover, acute cadmium ingestion can cause gastrointestinal irritation leading to GI tract erosion, abdominal pain, burning sensation, nausea, vomiting, salivation, and diarrhea (Jaishankar et al., 2014). It can also cause hepatic, renal, pulmonary damage, and coma. Chronic low-level exposure can cause cadmium to be deposited in the kidneys, leading to renal disease (Tchounwou et al., 2012). Data shows that the consumption of Cd through gluten-free foods is unlikely according to Inaba et al. (2006) as it would require 3,100 to 3,800 μg of Cd over a lifetime to cause mitochondrial dysfunction disease.

Cobalt (Co) levels ranged from 150 to 400 μg kg^−1^ ($\bar{x}$ = 252 μg kg^−1^) for all products including the U.S. market, Asian, and gluten containing foods. The U.S. market ranged from 150 to 400 μg kg^−1^ ($\bar{x}$ = 247), Asian ranged from 200 to 450 μg kg^−1^ ($\bar{x}$ = 275 μg kg^−1^), and gluten containing products at 200 μg kg^−1^ for the minimum, maximum and $\bar{x}$. Within the gluten-free foods (Asian / U.S. market), 4 samples were 1σ (i.e. 333 μg kg^−1^ Co) above the $\bar{x}$; (samples 14, 36, 41 [pastas], 23 [flour]). The highest Co was found in Asian gluten-free foods containing 200 to 450 μg kg^−1^ while the U.S. gluten-free foods contain between 200 and 450 μg kg^−1^. Most samples (53.2%) were within 4 to 140 μg kg^−1^ established by other researchers (Podio et al., 2013).

Cobalt is an essential micronutrient consumed in the form of vitamin B_12_. While this study indicates that Cr is at levels low enough as to not pose an acute effect unless a significant amount is consumed (Leyssens et al., 2017). Cobalt toxicity can occur acutely in large doses or cumulatively with long-term low level exposure according to Leyssens et al. (2017) and Simonsen et al. (2012). Water-soluble cobalt salts are rapidly absorbed from the small intestine. Co causes erythropoiesis by increasing erythropoietin production. It is also known for increasing the oxygen-carrying capacity of the blood and therefore enhancing physical endurance (Leyssens et al., 2017, Simonsen et al., 2012). Finally, it is low-level chronic exposure that can pose a toxicity issue to the consumer (Simonsen et al., 2012).

Chromium levels ranged from 100 to 1950 μg kg^−1^ ($\bar{x}$ = 575 μg kg^−1^) while the U.S. gluten free ranging between 100 and 550 μg kg^−1^; Asian foods ranged from 150 to 1950 μg kg^−1^ while gluten containing products varied from 1650 to 1800 μg kg^−1^. For Cr, no U.S. gluten-free were above the overall $\bar{x}$ of 575 μg kg^−1^; however, both the Asian and the gluten containing foods were above this level. Of the Asian and U.S. gluten-free foods, only 1 sample of a flour product (sample 39) was 1σ above the overall $\bar{x}$ (i.e. 575 μg kg^−1^). Current results exceed the literature established value by Tchounwou et al. (2012). It is important to point out that the highest Cr (1650 – 1800 μg kg^−1^) detected was in gluten containing products; twice that established in the literature.

The health hazards associated with Cr depend greatly on its oxidation state. This study considered Cr^3+^ and not the more harmful Cr^6+^due to its reduction to Cr^3+^ in a relatively short time and therefore, these foods would not contain the more harmful form of Cr^6+^ (Jaishankar et al., 2014). The most common forms are trivalent (Cr^3+^), a nutrient, and hexavalent (Cr^6+^), a known toxin (Jaishankar et al., 2014). Trivalent (III) Cr is readily oxidized to hexavalent (VI) due to the excess oxygen in the environment. Hexavalent (VI) Cr is highly water soluble, making it extremely toxic compared to the trivalent (III) form (Jaishankar et al., 2014, Tchounwou et al., 2012). Trivalent Cr is the typical form found in foods and usually not an issue. However, the uptake of Cr^6+^ by plants is known to happen; however, it is usually reduced by 96.3% to Cr^3+^ within 96 hours according to Shugaba et al. (2012).

Copper in gluten-free foods in U.S. products ranged between 600 and 2.25 × 10^4^ μg kg^−1^, Asian products ranged from 250 to 3800 μg kg^−1^, and gluten containing foods ranged from 3100 to 6100 μg kg^−1^ with an overall $\bar{x}$ of 4333 μg kg^−1^. Four samples (9, 11, 24, 28) were 1σ above the overall $\bar{x}$ (i.e. 4333 μg kg^−1^ Cu). However, the data is within established ranges (1800 to 1 × 10^4^ μg kg^−1^) according to Ertl and Goessler (2018) and the WHO (2004) maximum per day intake of 1 × 10^4^ μg kg^−1^ for ages 19 to 70 years. Copper is an essential nutrient as it serves as a co-factor for several oxidative stress-related enzymes (Kahn and Line (2010a, 2010b). It is also involved in the formation of hemoglobin, carbohydrate metabolism, catecholamine biosynthesis, and the formation of cross-links in collagen, elastin, and keratin in the hair (Tchounwou et al., 2012). The maximum exposure to Cu per day is recommended at 0.5 mg kg^−1^ according to the ATSDR (2008). Excess exposure to Cu leads to bioaccumulation in the liver, resulting in chronic hepatitis and cirrhosis according to Kahn and Line (2010a) and Tchounwou et al. (2012).

Iron concentrations in gluten-free products from the U.S. markets ranged from 100 to 6.32 × 10^4^ μg kg^−1^; Asian foods contained the lowest Fe (550 to 2.04 × 10^4^ μg kg^−1^); gluten containing foods ranged from 3.08 × 10^4^ to 5.02 × 10^4^ μg kg^−1^. Data is within the established limits (1.3 × 10^4^ to 1.03 × 10^5^ μg kg^−1^) for wheat and gluten-free food products as described by Ertl and Goessler (2018). Iron is an important nutrient as it is a vital cofactor for many proteins and enzymes; deficiency can lead to anemia and decreased oxygen delivery to tissues enzymes (Jaishankar et al., 2014, Kahn and Line, 2010b). Studies show that 20 mg kg-1 per day can lead to nausea, vomiting, and stomach pain, especially on low protein diets (Jaishankar et al., 2014).

Magnesium is another important part of a daily diet and vital for healthy functions. Mg however, was detected at 1.32 × 10^4^ to 1.96 × 10^6^ μg kg^−1^ with an overall $\bar{x}$ of 6.12 × 10^5^ μg kg^−1^. U.S. gluten-free foods were within the overall range, Asian products ranged between 2.54 × 10^4^ and 1.39 × 10^6^ μg kg^−1^ while gluten containing foods were between 2.44 × 10^5^ and 1.16 × 10^6^ μg kg^−1^ ($\bar{x}$ =5.49 × 10^5^ μg kg^−1^). Gluten-free products were 1σ above the overall $\bar{x}$ (i.e. 1.147 × 10^6^ μg kg^−1^ Fe) in 14% of samples (9, 11, 18, 24, 28, 34). Findings are comparable with other studies (350 to 1.4 × 10^4^ μg kg^−1^) as provided by Podio et al. (2013). The risk of magnesium toxicity is increased in individuals with decreased renal function or with renal failure as the kidney is responsible for removing excess magnesium from the body (Higdon et al., 2014). The Food and Nutrition Board (FNB) Institute of Medicine (1997), and Ertl and Goessler (2004) state that consuming 3 × 10^5^ and 4.2 × 10^5^ per day is healthy and therefore, findings are within established norms.

Manganese detected in U.S. foods ranged from 400 to 8.95 × 10^4^ μg kg^−1^ with an overall $\bar{x}$ of 2.78 × 10^4^ μg kg^−1^. Asian foods ranged from 400 to 2.09 × 10^4^ μg kg^−1^ while gluten samples ranged from 1.5 × 10^4^ to 4.83 × 10^4^ μg kg^−^1. Samples contained Mn at 1σ above the overall $\bar{x}$ in 14% of the gluten-free samples (9, 11, 18, 24, 28, 34, and 45). Manganese is an essential nutrient, but can have toxic consequences if too much is ingested. The primary oral route for intoxication is through contaminated water as its bioavailability is higher in water (Higdon et al., 2014). The risk of manganese neurotoxicity is set at 2mg/day for children 1–3 years; 3mg/day for children 4–8 years; 6mg/day for children 9–13 years; 9mg/day for adolescents 14–18 years, and 11mg/day for adults >19 years (O’Neal and Zang, 2015). These findings are analogous with other published works (350 to 1.4 × 10^4^ μg kg^−1^) for Mn in gluten-free and gluten containing foods (Podio et al., 2013). If a person has a Fe deficient diet, there is an increased risk of Mn toxicity as Fe deficiency enhances the gastrointestinal absorption of Mn, leading to possible cognitive deficits (Higdon et al., 2014, FNB, 1997).

Molybdenum was detected in 78.7% of foods with a range of 250 and 6100 μg kg^−1^ with an overall $\bar{x}$ of 1294 μg kg^−1^. Gluten-free products (U.S. markets) ranged between 250 and 6100 μg kg^−1^, Asian products ranged from 350 to 2100 μg kg^−1^, and gluten containing foods ranged between 700 and 1050 μg kg^−1^. Data show that 9.5% of samples were 1σ above the overall $\bar{x}$ (2580 μg kg^−1^) for pasta gluten-free foods (samples 2, 5, 6, 22). Molybdenum toxicity is relatively rare in humans as it functions as a co-factor in four Mo-dependent enzymes necessary for health (Higdon et al., 2014, O’Neal and Zang, 2015). Other studies have set a daily intake of 300 μg /day for children 1–3 years; 600 μg /day for children 4–8 years; 1.1mg/day for children 9–13 years; 1.7mg/day for adolescents 14–18 years, and 2.0mg/day for adults >19 years (O’Neal and Zang, 2015, Suchowilska et al., 2012). Although the Institute of Medicine found little evidence of adverse health effects related to excess Mo consumption in healthy people, they have set a daily UIL at 300 μg /day for children 1–3 years, 600 μg /day for children 4–8 years, 1.1 mg/day for children 9–13 years, 1.7 mg/day for adolescents 14–18 years, and 2.0 mg/day for adults >19 years (Higdon et al., 2014). Levels are within limits set in other studies (420 to 580 μg kg^−1^ Mo) for gluten-free and gluten containing foods.

Nickel was detected at 150 to 5000 μg kg^−1^ with an overall $\bar{x}$ of 1757 μg kg^−1^. Gluten-free products from the U.S. ranged between 150 and 4350 μg kg^−1^, Asian foods ranged from 300 4350 μg kg^−1^, and gluten containing foods ranged between 4950 and 5350 μg kg^−1^. In 14.3% of samples, Ni was 1σ above the overall $\bar{x}$ (1757 μg kg^−1^) across a mix of pasta and flour food products (samples 5, 9, 14, 23, 41, 42). Additionally, four gluten containing pastas (samples 43–45, 47) were 1σ above the overall $\bar{x}$. In the literature, Ni detected in foods is normally lower than 1000 μg kg^−1^ as described by Ekholm et al. (2007); placing these finding above acceptable ranges in 33% of gluten-free foods and 60% of gluten containing foods. The risk of Ni toxicity is low due to its limited intestinal absorption according to ATSDR (2008).

Titanium ranged from 7000 to 3.1 × 105 μg kg^−1^ with an overall $\bar{x}$ of 5.53 × 104 μg kg^−1^. Gluten-free products (U.S. market) ranged from 7000 and 2.17 × 105 μg kg^−1^, Asian products ranged between 8350 and 3.1 × 105 μg kg^−1^, and gluten containing foods ranged between 1.03 × 104 and 3.62 × 104 μg kg^−1^. Compared with other elements, Ti was found to be high and more in line with crustal metals (i.e. Ca, Mg) with 14.3% of the samples (7, 14, 23, 26, 36, 41) 1σ above the overall $\bar{x}$ for gluten-free pastas and flours. Other authors have determined that the typical exposure to food-grade TiO_2_ in an average adult to be on the order of 1000 μg kg^−1^ per day (Weir et al., 2012); placing this data above the established norms in 100% of samples. It is important to note that Weir et al. (2012) stated there is an important difference between total Ti (_T_Ti), TiCl_4_’ and food-grade TiO_2_. Other researchers have shown that TiCl_4_ is the harmful form of Ti; however, the forms of Ti found in foods are TiO_2_ MPs (micro-particles) and TiO_2_ NPs (nanoparticles) thus, no limit is specified in the literature at this time (ATSDR, 2008).

Zinc ranged from 5350 to 8.0 × 104 μg kg^−1^ with an overall $\bar{x}$ of 3.9 × 104 μg kg^−1^ in analyzed foods. Gluten-free (U.S. market) ranged between 5750 and 8.0 × 104 μg kg^−1^; Asian products ranged between 5350 and 3.7 × 104 μg kg^−1^; while gluten containing foods ranged from 2.8 × 104 and 6.8 × 104 μg kg^−1^. Zinc was also in line with crustal metals (i.e. Ca, Mg) with 23.8% (samples 5, 6, 9, 11, 14, 15, 22–24, 28) 1σ above the overall $\bar{x}$ (6.26 × 104 μg kg^−1^) with sample 45 (gluten containing pasta) significantly greater than 1σ of the overall $\bar{x}$. Other studies have determined that the average Zn in foods range between 9400 and 5 × 104 μg kg^−1^ (Ertl and Coessler, 2018). It is known that Zn is an important trace element to the human diet and is therefore required as part of our food chain (Bermudez et al., 2011). The levels of Zn that can produce an adverse health effect are much higher than the Recommended Dietary Allowances (RDAs) of 11 mg/day for men and 8 mg/day for women (ATSDR, 2008). Likewise, data places Zn in the gluten-free foods above the established range in 35.7% in the gluten-free samples and 20% of gluten containing foods. Therefore, the levels of Zn that can produce an adverse health effect are much higher than the Recommended Dietary Allowances (RDAs) of 11 mg/day for men and 8 mg/day for women which this data is below (ATSDR, 2008).

## 4. Conclusions

This study produced similar results to those of Orecchio et al. (2014, 2015) where they evaluated trace elements in gluten-free foods across Italy. Most elements were detected in all samples with Ca, Fe, Mg, Ti and Zn being those with the greatest $\bar{x}$ concentrations across food products but, below harmful levels with a normal, healthy diet. Cadmium was detected (0–600 μg kg^−1^) in 96.8% of the U.S., 54.5% of Asian gluten-free foods, and in 100% of gluten containing foods, although all below action limits in the literature (2004). Cobalt was present in 48.4 % of the U.S., 72.7% of Asian gluten-free foods, and 40% of the gluten containing foods; Cr was detected in 54.8% of the U.S., 72.7% of Asian gluten-free foods, and 100% of the gluten containing products. Chromium in gluten-free foods was below reported averages of 799 μg kg^−1^ except in sample 39 and all of the gluten containing foods; however, even these levels are below the action limits reported by the WHO (2004). There was little difference in trace metal content observed between the pasta or flour products however, gluten containing food had higher levels of metals then those deemed gluten free.

It has been suggested that gluten-free foods may at times contain higher levels of metals than gluten containing foods (Orecchio et al., 2014, Thompson, 2000). This study found elevated trace metals were more commonly found in the gluten containing products, but at limits below WHO (2004) action levels. This is likely the product used for gluten free (i.e. rice) do not have an affinity to up-take metals as does other products such as wheat and barely. It is possible that low-level exposure to metal laden gluten-free and gluten containing foods, even at low levels, can lead to chronic toxicity with long-term consumption. Additional research is required to better understand the sources and pathways of trace elements in these foods.

## Figures and Tables

**Table 1.** Data covering overall pasta and flour product data set: United States Market, Asian Market, and Gluten foods

Source	Type	ID	Al	Ba	Ca	Cd	Co	Cr	Cu	Fe	Mg	Mn	Mo	Ni	Ti	Zn
US market	Pasta-r	**1**	1400	600	375400	150	-	-	600	100	13250	400	-	200	30200	5950
US market	Pasta-r	**2**	1950	1750	332300	200	150	100	7300	39600	739000	20850	4600	1000	36950	57100
US market	Pasta-r	**3**	2800	2650	198200	250	-	200	3500	27450	842500	43300	1050	500	96350	54600
US market	Pasta-r	**4**	550	1200	109400	150	150	150	2500	9250	804000	58100	900	300	13650	43250
US market	Pasta-r	**5**	1050	3600	274750	400	150	550	7500	54300	756500	25750	6100	3600	30800	70000
US market	pasta-r	**6**	2350	1950	453050	350	200	200	6650	42750	864500	35050	2650	750	54200	75350
US market	Pasta-r	**7**	6750	2800	618500	200	250	200	3150	26700	704500	32950	400	500	124000	40300
US market	Pasta-r	**8**	700	1150	82650	300	-	-	2050	6850	631000	46700	1000	350	20250	36100
US market	Flour-r	**9**	550	750	303250	200	-	550	18500	57100	1817500	83100	450	4050	14800	64250
US market	Flour-r	**10**	400	1450	253700	-	-	-	-	-	28950	1050	-	150	7000	5750
US market	Flour-r	**11**	600	1100	273200	250	300	400	22550	63200	1871000	74600	750	1850	82600	79550
US market	Flour-r	**12**	850	1550	455900	150	-	-	1250	14750	258050	1900	1200	600	35200	13300
US market	Flour-r	**13**	650	600	402200	150	-	100	2900	1650	974610	2850	250	150	32500	15750
US market	Pasta-r	**14**	2950	7100	856000	200	400	200	7650	46800	984000	63300	1650	3850	162400	77150
US market	Pasta-r	**15**	2150	1300	246300	200	300	-	7100	39800	669000	25000	1350	1800	57550	69550
US market	Pasta-r	**16**	400	650	42400	350	-	-	750	7400	202000	3000	500	550	51700	14500
US market	Pasta-r	**17**	950	1900	104300	300	300	-	1950	7250	365550	22800	900	400	24450	38950
US market	Pasta-r	**18**	2050	1650	176700	350	150	200	3150	28650	1223000	79900	850	1000	42200	52400
US market	Pasta-r	**19**	1050	6800	277050	150	-	-	1950	7200	312600	13150	600	950	22700	24000
US market	Pasta-r	**20**	1150	1600	128650	250	-	-	2550	8700	850000	54100	750	250	17200	47600
US market	Pasta-r	**21**	1100	1750	107750	400	-	200	2450	9650	687000	39850	1250	500	50050	51850
US market	Pasta-r	**22**	2550	2500	237100	250	250	-	7550	52100	590500	19800	4500	2100	59750	66400
US market	Flour-r	**23**	5900	12100	840000	200	350	150	8600	49600	1138000	89500	1750	4350	217800	77300
US market	Flour-r	**24**	1250	3450	2094500	250	250	300	9700	32100	1967500	36300	550	800	39800	66750
US market	Flour-r	**25**	900	500	65100	300	-	-	1800	-	158100	15100	400	450	24500	25800
US market	Flour-r	**26**	1100	1550	14300	450	-	150	2350	12350	614500	22050	900	850	120100	34800
US market	Flour-r	**27**	3250	800	96700	600	250	-	2300	34900	865000	29200	1100	2300	86400	50050
US market	Flour-r	**28**	1950	9000	1436500	300	250	250	13850	46000	1804500	69900	300	2100	55250	80400
US market	Flour-r	**29**	1050	650	1936000	250	-	-	1100	49200	276300	26300	750	300	19900	25500
US market	Flour-r	**30**	900	950	188600	200	-	-	1200	5300	370050	23500	250	400	24350	23350
US market	Flour-r	**31**	1700	1900	191600	350	-	200	2250	13350	593500	19400	1200	900	85700	37850
Asian Market	Pasta-r	**32**	950	1300	65000	500	-	-	3500	3550	121550	20900	900	300	20200	30300
Asian Market	Flour-r	**33**	650	3450	336050	-	200	-	300	550	56900	8550	2100	-	11100	6400
Asian Market	Flour-r	**34**	2400	450	137550	-	200	200	3800	20450	1392500	18650	-	500	8350	37900
Asian Market	Flour-r	**35**	1600	1200	100800	250	200	650	1000	2000	115300	14400	1750	350	30650	30550
Asian Market	Pasta-r	**36**	2150	2200	118400	-	350	300	1600	4050	42350	4800	850	2150	148650	26450
Asian Market	Pasta-r	**37**	3000	4050	141650	250		400	850	7200	86900	3850	350	1850	94850	16550
Asian Market	Flour-r	**38**	950	1100	145100	300	200	150	1300	1400	129700	13000	-	-	26450	27700
Asian Market	Pasta-r	**39**	1950	350	109100	-	250	1950	-	6700	31700	400	-	500	24350	5350
Asian Market	Pasta-r	**40**	550	650	194800	-	350	-	-	-	33300	-	-	-	11050	6750
Asian Market	Pasta-r	**41**	2000	850	84300	450	450	300	250	2650	39550	1550	-	5600	311550	10200
Asian Market	Pasta-r	**42**	2900	650	115800	250	-	700	300	4700	25400	1000	-	5900	59350	7900
Gluten Foods	Pasta-w	**43**	1450	4750	209300	250	-	1700	3700	34950	433500	19850	700	5350	10350	28000
Gluten Foods	Pasta-w	**44**	1600	5250	207750	250	200	1700	3150	33650	457800	15400	1050	4950	22050	47850
Gluten Foods	Pasta-w	**45**	2150	9700	323100	250	200	1800	5000	43750	1168000	48300	-	5100	22350	68050
Gluten Foods	Pasta-w	**46**	1850	5550	571500	200	-	1650	6100	50200	244850	15000	-	-	22550	29300
Gluten Foods	Pasta-w	**47**	3350	3700	659000	350	-	1650	3100	30800	441950	18550	-	5150	36250	47450

NOTE: Data is reported in ug kg^−1^. Sample Identification (ID). When data is less than instrument detection level (IDL), it is represented as a “- “. Sample data to be valid must be above limit of quantitation (LOQ). Statistical data shown is broken into four (4) areas; overall data, United States market gluten free foods (USM), Asian gluten free foods (Asian), and gluten containing foods (control), source of food type: r = rice products, w = wheat products

**Table 2.** Statistical data covering overall data set, United States products (USM), Asian products (Asian), and Gluten containing products (Control)

Source	Statistical parameter	Al	Ba	Ca	Cd	Co	Cr	Cu	Fe	Mg	Mn	Mo	Ni	Ti	Zn
	σ	1275	2628	438831	101	81	614	4645	19507	535204	24212	1286	1824	58792	23223
Overall	$\bar{x}$	1754	2606	355133	278	252	575	4333	23651	612717	27890	1294	1757	55328	39407
data	**MIN**	400	350	14300	150	150	100	250	100	13250	400	250	150	7000	5350
	**MAX**	6750	12100	2094500	600	400	1950	22550	63200	1967500	89500	6100	5900	311550	80400
	**IDL**	5	2	5	2	2	2	2	3	5	3	3	3	3	5
	**LOQ**	15	6	15	6	6	6	6	10	15	10	10	10	10	15
		Al	Ba	Ca	Cd	Co	Cr	Cu	Fe	Mg	Mn	Mo	Ni	Ti	Zn
	σ	1465	2685	515270	104	77	136	5276	19746	524697	25700	1406	1234	47479	23186
USM	$\bar{x}$	1708	2494	424905	270	247	241	5223	27381	773434	34798	1341	1221	56139	45982
	**MIN**	400	500	14300	150	150	100	600	100	13250	400	250	150	7000	5750
	**MAX**	6750	12100	2094500	600	400	550	22550	63200	1967500	89500	6100	4350	217800	80400
	σ	867	1240	73306	113	96	587	1342	5733	401170	7578	715	2335	91634	12044
Asian	$\bar{x}$	1736	1477	140777	333	275	581	1433	5325	188650	8710	1190	2144	67868	18732
	**MIN**	550	350	65000	250	200	150	250	550	25400	400	350	300	8350	5350
	**MAX**	3000	4050	336050	500	450	1950	3800	20450	1392500	20900	2100	5900	311550	37900
	σ	758	2296	209500	55	-	61	1305	8058	356646	14060	247	165	9175	16410
Control	$\bar{x}$	2080	5790	394130	260	200	1700	4210	38670	549220	23420	875	5138	22710	44130
	**MIN**	1450	3700	207750	200	200	1650	3100	30800	244850	15000	700	4950	10350	28000
	**MAX**	3350	9700	659000	350	200	1800	6100	50200	1168000	48300	1050	5350	36250	68050

NOTE: Data is reported in ug kg^−1^. When data is less than instrument detection level (IDL), it is represented as a “ - “. Sample data to be valid must be above limit of quantitation (LOQ). Statistical data shown is broken into four (4) areas; overall data, United States market gluten free foods (USM), Asian gluten free foods (Asian), and gluten containing foods (control); mean: $\bar{x}$; standard deviation: σ
